# A Complete Study of Farrerol Metabolites Produced In Vivo and In Vitro

**DOI:** 10.3390/molecules24193470

**Published:** 2019-09-24

**Authors:** Jintuo Yin, Yinling Ma, Caijuan Liang, Hairong Wang, Yupeng Sun, Lantong Zhang, Qingzhong Jia

**Affiliations:** 1 Department of Pharmaceutical Analysis, School of Pharmacy, Hebei Medical University, Shijiazhuang 050017, China; yinjintuo@163.com (J.Y.); caijuanliang@126.com (C.L.); wel_lw@163.com (H.W.); yupengsun@hebmu.edu.cn (Y.S.); 2 National Clinical Drug Monitoring Center, Department of Pharmacy, Hebei Province General Center, Shijiazhuang 050051, China; 3 Department of Pharmacology, Hebei Medical University, Shijiazhuang 050017, China

**Keywords:** farrerol, UHPLC-Q-TOF-MS/MS, multiple data post-processing techniques, metabolism, in vivo, in vitro

## Abstract

Although farrerol, a characteristically bioactive constituent of *Rhododendron dauricum* L., exhibits extensive biological and pharmacological activities (e.g., anti-oxidant, anti-immunogenic, and anti-angiogenic) as well as a high drug development potential, its metabolism remains underexplored. Herein, we employed ultra-high performance liquid chromatography/quadrupole time-of-flight mass spectrometry coupled with multiple data post-processing techniques to rapidly identify farrerol metabolites produced in vivo (in rat blood, bile, urine and feces) and in vitro (in rat liver microsomes). As a result, 42 in vivo metabolites and 15 in vitro metabolites were detected, and farrerol shown to mainly undergo oxidation, reduction, (de)methylation, glucose conjugation, glucuronide conjugation, sulfate conjugation, *N*-acetylation and *N*-acetylcysteine conjugation. Thus, this work elaborates the metabolic pathways of farrerol and reveals the potential pharmacodynamics forms of farrerol.

## 1. Introduction

**Figure 1:**
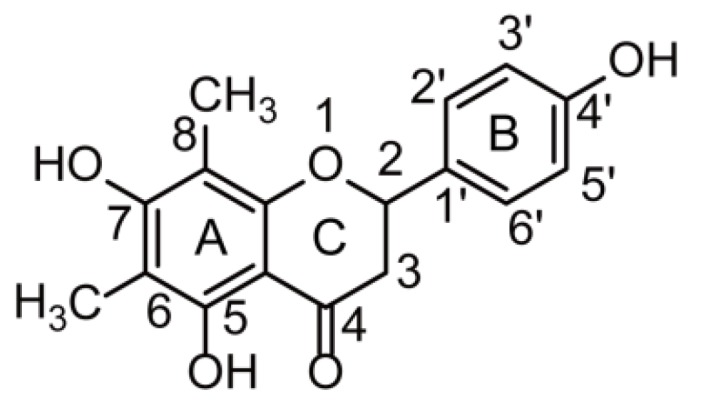
The chemical structure of farrerol.

The term “flavonoids” refers to a broad category of plant polyphenolics that are found in fruits, vegetables, beverages, and traditional Chinese medicines [1,2,3], and exhibit potent anti-oxidant, anti-inflammatory, anti-cancer, anti-bacterial, anti-viral, anti-allergic, and neuroprotective activities [3,4,5,6,7]. The leaves of *Rhododendron dauricum* L. (RD), a plant used as a traditional Chinese medicine, are rich in flavonoids, some of which exhibit anti-inflammatory, anti-bacterial, and anti-oxidant activities [8]. Farrerol (Figure 1) is a natural bioactive constituent of RD with extensive biological and pharmacological (e.g., anti-inflammatory, anti-bacterial, anti-oxidant, anti-immunogenic, and anti-angiogenic) activities [9,10,11,12,13] and a high potential to be developed into a natural therapeutic agent for the treatment of colitis, lung cancer, gastric cancer, and cardiovascular diseases [14,15,16,17]. Although numerous studies have probed the pharmacological activity of farrerol, its metabolism has not been investigated, which hinders the further exploration of farrerol biological activity and clinical therapeutic effect. Thus, a study on farrerol metabolite identification is expected to facilitate the development of new pharmaceuticals and provide new insights into the corresponding pharmacological mechanism of action.

Drug metabolism refers to the enzyme-catalyzed biotransformations of a drug administered to the body and often affords metabolites with superior pharmacological activity [18] or potential toxicity [19]. Hence, a study of farrerol metabolism is necessary to explore the biotransformations of this flavonoid and ensure its safe use. Only glucuronide conjugation metabolites of farrerol have previously been detected in rat urine [20], and no systematic data on farrerol metabolism in rats are available. Additionally, liver microsomes are often used to mimic in vivo metabolism because of their crucial metabolizing enzymes responsible for drug biotransformations [20]. In view of the above, we herein studied the metabolism of farrerol in rats (blood, bile, urine and feces) after oral administration and rat liver microsomes by incubation to understand the safety and efficacy of the corresponding metabolites and explore the possibilities of new drug discovery.

Ultra-high-performance liquid chromatography coupled with tandem mass spectrometry (UHPLC-MS/MS) is widely employed for the rapid and accurate qualitative analysis of biological samples [21], as exemplified by the popularity of using ultra-high-performance liquid chromatography coupled with quadrupole time-of-flight mass spectrometry (UHPLC-Q-TOF-MS/MS) for metabolite identification [21,22]. Therefore, these popular technologies were herein chosen to probe farrerol metabolism. Information-dependent acquisition (IDA) patterning was employed to acquire accurate MS and MS/MS data of metabolites by UHPLC-Q-TOF-MS/MS [23], and MetabolitePilot 2.0 software with multiple post-processing functions (extracted ion chromatogram (XIC), mass defect filtering (MDF), product ion filtering (PIF), and neutral loss filtering (NLF)) was employed for metabolite identification and characterization [24].

As a result, a rapid and efficient method was established to identify and characterize farrerol metabolites produced in vivo (42 metabolites) and in vitro (15 metabolites), which allowed us to summarize farrerol metabolic pathways for the first time. Thus, this work provides detailed information on farrerol metabolism, shows that the combined application of UHPLC-Q-TOF-MS/MS and post-processing techniques is a feasible strategy for food and drug metabolic studies, and lays the foundation for related drug discovery and pharmacological mechanism research.

## 2. Results

### 2.1. Analytical Procedure

**Figure 2:**
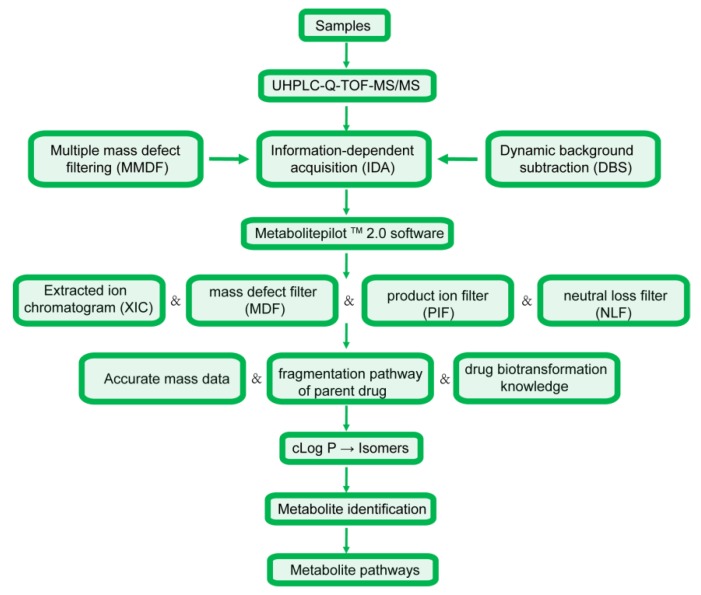
The workflow for the metabolic study.

The analysis strategy was divided into four steps, with the corresponding flow chart presented in Figure 2. (1) Sample collection, treatment and data acquisition: online full-scan data were acquired relying on multiple mass defect filtering and dynamic background subtraction to obtain accurate MS and MS/MS spectra in the IDA pattern [23], and metabolite identification was then carried out using MetabolitePilot 2.0 software and numerous data post-processing operations [23,24]. (2) Data analysis: among these operations, XIC and MDF were employed to predict metabolite molecular weights and elemental compositions based on accurate MS data [25], while PIF and NLF were employed to detect metabolites with fragmentation pathways similar to those of the parent drug [25]. (3) Metabolic interpretation: accurate mass datasets, parent drug cleavage patterns, and related drug biotransformation information were used to describe metabolic processes [23]. (4) As an important parameter, cLog P values predicted by ChemDraw 14.0 were used to discriminate between metabolite isomers [25,26], as metabolites with larger cLog P values are usually eluted more slowly in reverse-phase chromatographic systems [23].

### 2.2. Farrerol Fragmentation Patterns

**Figure 3:**
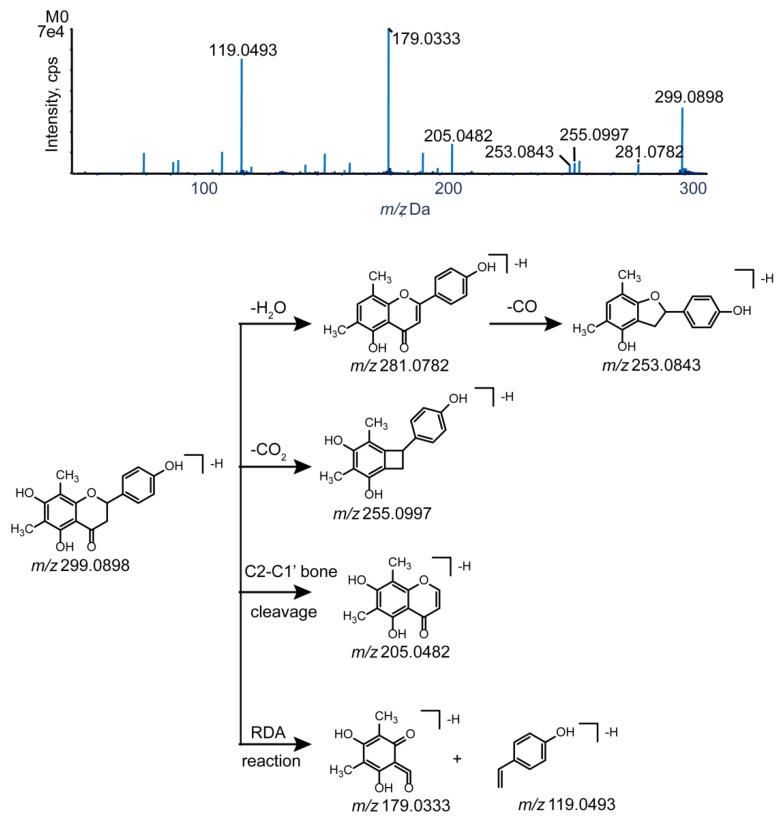
The MS/MS spectrum and the cleavage pathways of farrerol.

Knowledge of the parent drug fragmentation patterns is crucial for metabolite identification. To obtain extensive information on farrerol fragment ions, the standard solution of farrerol was analyzed by UHPLC-Q-TOF-MS/MS in negative-ion electrospray ionization (ESI) mode. The parent drug (farrerol, M0) with the molecular formula of C_17_H_16_O_5_ was eluted at 12.60 min with high intensity and yielded a deprotonated ion with *m/z* 299.0898. The corresponding ESI-MS^2^ spectrum featured product ions with *m/z* 281.0782, 255.0997, and 253.0843 produced by loss of H_2_O, CO_2_, and H_2_O + CO, respectively, while the product ion with *m/z* 205.0483 was formed through C2–C1′ bond cleavage. Additionally, secondary ions with *m/z* 179.0333 and 119.0493 were produced by retro-Diels−Alder (RDA) cleavage of ring C, and this reaction was concluded to be a representative flavonoid fragmentation pathway allowing rapid metabolite identification [21,27,28]. The MS/MS spectra and fragmentation pathways of farrerol are presented in Figure 3.

### 2.3. Identification of Phase I Metabolites

**Figure 4:**
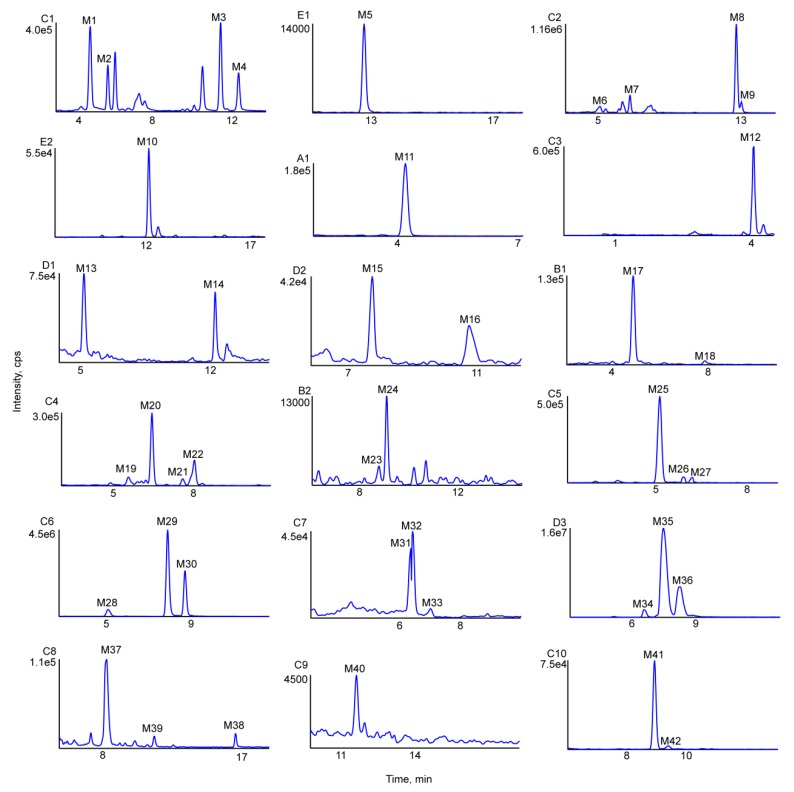
Extracted ion chromatograms of all metabolites of farrerol in vivo and in vitro (A1-in rat plasma, B1-2 in rat bile, C1-10 in rat urine, D1-D3 in rat feces, E1-2 in rat liver microsomes).

**Figure 5:**
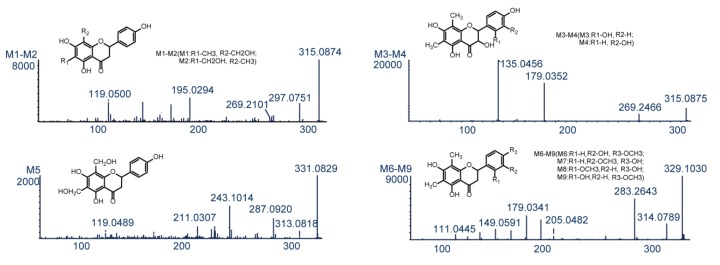

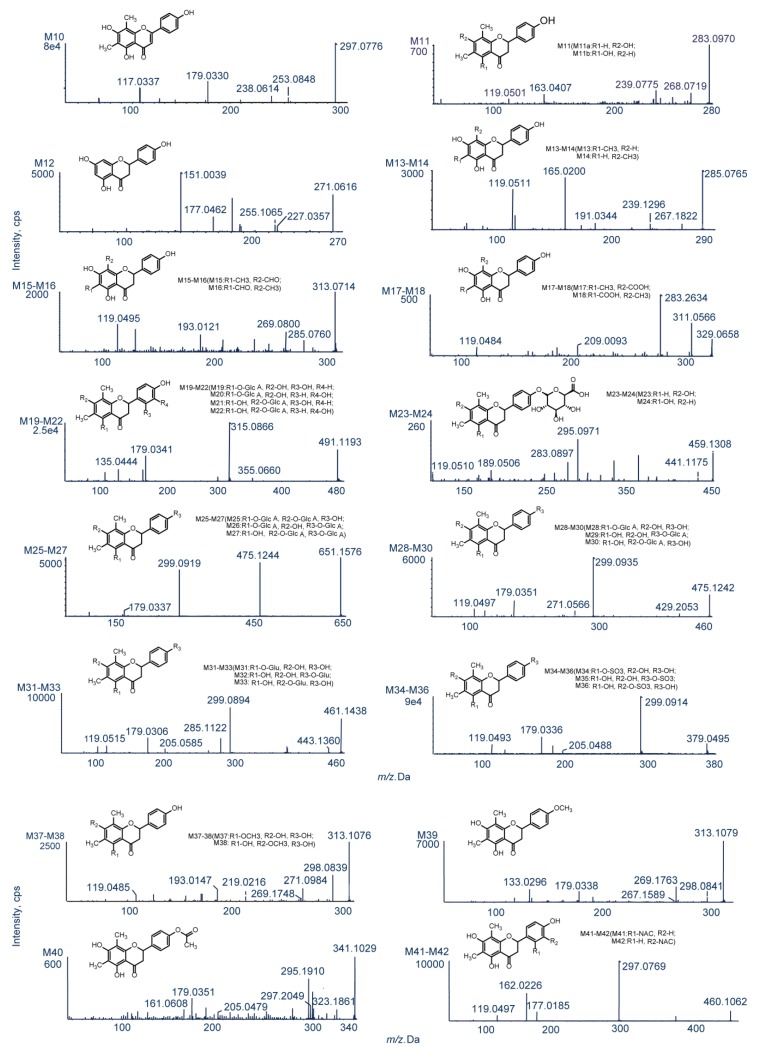
The chemical structures and MS/MS spectra of metabolites of farrerol.

UHPLC-MS was used to compare in vivo samples (obtained after oral administration) and in vitro samples (obtained after incubation of liver microsomes) to blank samples for the purpose of metabolite identification. As a result, 42 in vivo metabolites and 15 in vitro metabolites were identified in negative-ion ESI mode, and metabolic reactions in vivo were found to be more complex than those in vitro. The obtained metabolite information is presented in Table 1. Figure 4 shows the extracted ion chromatograms of farrerol metabolites in blood, bile, urine, feces and liver microsome samples, and Figure 5 shows their chemical structures and MS/MS spectra.

#### 2.3.1. Oxidation Reactions

Metabolites M1–M4 (C_17_H_16_O_6_, error ≤ 0.6 ppm) were eluted at 4.75, 5.69, 11.65, and 12.62 min, and yielded deprotonated ions with *m/z* 315.0874, 315.0874, 315.0875, and 315.0879, respectively. These ions were 16 Da (O) heavier than the corresponding ion produced from farrerol, and this mass difference was hereinafter denoted as Δ, i.e., Δ = 16 in this case. Typical product ions observed for M1–M4 with *m/z* 297 [M−H−H_2_O]^−^ and 269 [M−H−H_2_O−CO]^−^ manifested that these metabolites were produced by oxidation [23]. Fragment ions with *m/z* 195.0294 and 119.0500 detected for M1 and M2 were produced by the RDA reaction. The ion with *m/z* 195.0294 had Δ = 16 Da (O), i.e., oxidation occurred at the 6-CH_3_ or 8-CH_3_ positions of ring A. However, M1 and M2 had equal cLog P values (1.75585) and could not be discriminated. The ESI-MS^2^ spectra of M3 and M4 featured two prominent product ions with *m/z* 179.0352 and 135.0456, attributable to the RDA reaction [28]. In the same way, the ion with *m/z* 135.0456 had Δ = 16 Da (O), which indicated that oxidation took place at position 2′ or 3′ of ring B. The cLog P values of M3 (2.57585) and M4 (2.69585) allowed these compounds to be identified as 2′- and 3′-hydroxyfarrerol, respectively.

M5 (retention time = 12.84 min) afforded a deprotonated ion with *m/z* 331.0821 and had an elemental composition of C_17_H_16_O_7_ (error = 1.8 ppm), which corresponded to the addition of two oxygens to farrerol. The ESI-MS^2^ spectrum of M5 featured two dominant product ions with *m/z* 313.0818 and 287.0920, generated through the loss of H_2_O and CO_2_, respectively [29]. Moreover, typical product ions of the RDA reaction were observed at *m/z* 211.0307 and 119.0489 [29]. The ion with *m/z* 211.0307 had Δ = 32 Da (2O), which indicated that both oxidation sites were on ring B. Based on the above, M5 was identified as A-ring-dihydroxylated farrerol.

M6–M9 had a molecular formula of C_18_H_18_O_6_ (error ≤ 2.9 ppm), were eluted at 5.04, 6.70, 12.78, and 13.06 min, and yielded deprotonated ions with *m/z* 329.1030, 329.1029, 329.1040, and 329.1031, respectively (Δ = 30 Da (CH_2_O)). As ions with *m/z* 314.0789 and 283.2643 were produced by loss of CH_3_ and H_2_O + CO, respectively, M6–M9 were concluded to be formed by oxidation and methylation. Ions with *m/z* 205.0482 and 179.0341 were generated through C2–C1′ bond cleavage and the RDA reaction, respectively, in agreement with the corresponding transformations of farrerol. The RDA reaction–produced fragment ion with *m/z* 149.0591 had Δ = 30 Da (O + CH_2_), which indicated that both oxidation and methylation (a total of four cases) occurred at ring B. Based on their cLog P values (3.14205, 3.14205, 3.34205, and 3.39205, respectively), M6–M9 were identified as 3′-methoxyfarrerol, 3′-hydroxy-4′-methoxyfarrerol, 2′-hydroxy-4′-methoxyfarrerol, and 3′-methoxyfarrerol, respectively.

M10 was eluted at 12.20 min and yielded a deprotonated ion with *m/z* 297.0776, Δ = −2 Da (2H). The two characteristic product ions with *m/z* 253.0848 and 238.0614 were produced by successive loss of CO_2_ and CH_3_ [28]. Ions with *m/z* 209.0946 and 179.0330 were generated by C2–C1′ bond cleavage and the RDA reaction, respectively, which was consistent with the fragmentation pattern of farrerol. The diagnostic ion with *m/z* 117.0337 produced by the RDA reaction had Δ = −2 Da (2H) [30]. Based on the above, M10 was tentatively identified as 2,3-di-dehydrofarrerol (C_17_H_14_O_5_, error = 2.6 ppm).

#### 2.3.2. Loss of O (deoxygenation)

M11 was eluted at 4.20 min and afforded a deprotonated ion with *m/z* 283.0970 (Δ = −16 Da). Product ions with *m/z* 268.0719 and 239.0775 were ascribed to loss of CH_3_ and CO_2_, respectively [28]. The RDA reaction afforded prominent ions with *m/z* 163.0407 and 119.0501. The former ion had Δ = −16 Da (O), which implied the loss of a hydroxyl group at C5 or C7 of ring A [31]. The molecular formula of M11 was determined as C_17_H_16_O_4_ (error = −2 ppm), with the two plausible structures corresponding to this formula shown in Figure 5.

#### 2.3.3. Demethylation

M12 (C_15_H_12_O_5_, error = 1.6 ppm) was eluted at 4.46 min and afforded a deprotonated ion with *m/z* 271.0616 (Δ = −28 Da (2CH_2_)). Distinctive secondary ions with *m/z* 227.0357 and 225.1065 were produced through the loss of CO_2_ and CO + H_2_O, respectively [23]. The product ion with *m/z* 177.0462 produced by C2–C1′ bond cleavage had Δ = −28 Da (2CH_2_), which was indicative of bis-demethylation. Another typical fragment ion with *m/z* 119.0501 was identified and was ascribed to the loss of two methyl groups on ring A. Based on the above, M12 was definitively recognized as 6,8-bis-demethylated farrerol, also known as naringenin.

M13 (eluted at 5.38 min, cLog P = 2.84385) and M14 (eluted at 12.24 min, cLog P = 2.89385) yielded deprotonated ions with *m/z* 285.0765 and 285.0768 (Δ = −14 Da (CH_2_)), respectively, and were assigned the molecular formula of C_16_H_14_O_5_ (error ≤ −0.1 ppm). Prominent ions with *m/z* 267.1822 and 239.1296 were produced by successive loss of H_2_O and CO [23]. Two key product ions with *m/z* 165.0200 (Δ = −14 Da (CH_2_)) and 119.0511 were produced through RDA cleavage [29]. Based on the above, M13 and M14 were concluded to be products of demethylation and were identified as 8-demethylated farrerol and 6-demethylated farrerol, respectively.

M15 and M16 (C_17_H_14_O_6_, error ≤ ±1.3 ppm, cLog P = 2.50612 and 2.92612, respectively) were eluted at 7.87 and 10.85 min and afforded deprotonated ions with *m/z* 313.0714 and 313.0723 (Δ = 14 Da), respectively. The two major product ions with *m/z* 285.0760 and 269.0800 were ascribed to loss of CO and CO_2_, respectively [30]. RDA cleavage afforded ions with *m/z* 193.0121 (Δ = 14 Da) and 119.0495 [21]. Based on the above, M15 and M16 were identified as 8-ketofarrerol and 6-ketofarrerol, respectively.

The ESI-MS^2^ spectra of M17 and M18 featured deprotonated ions with *m/z* 329.0658 and 329.0662 (Δ = 30 Da), respectively, as well as ions with *m/z* 311.0566 and 283.2634 formed by successive loss of H_2_O and CO [29]. The presence of RDA reaction–produced ions with *m/z* 209.0093 and 119.0484 indicated that M17 and M18 were de-methylated to carboxylic acid metabolites. M17 and M18 were respectively eluted at 4.89 and 7.90 min and possessed the same chemical formula of C_17_H_14_O_7_ (error ≤ −1.4 ppm), as determined by XIC and MDF. Based on the above, M17 (cLog P = 2.86826) and M18 (cLog P = 3.54826) were identified as 8-acidized farrerol and 6-acidized farrerol, respectively.

### 2.4. Identification of Phase II Metabolites

M19–M22 (eluted at 5.57, 6.46, 7.62, and 8.09 min, respectively) were isomers with the formula C_23_H_24_O_12_ (error ≤ −0.1 ppm). The respective deprotonated ions had *m/z* 491.1193, 491.1192, 491.1195, and 491.1194 [176 Da (C_6_H_8_O_6_) heavier than those produced from M1–M4], which was indicative of oxidation and glucuronide conjugation. In addition, the RDA reaction–produced prominent ion with *m/z* 355.0660 was 176 Da (C_6_H_8_O_6_) heavier than the ion with *m/z* 179.0333, which indicated that the glucuronide moiety was linked to ring A. Another typical ion with *m/z* 179.0341 was generated by loss of C_6_H_8_O_6_ based on the ion *m/z* 355.0660. The observation of an RDA reaction-produced ion with *m/z* 135.0444, similarly to the case of M3/M4, suggested that the oxidation sites were on ring B. Based on the above and the respective cLog P values of 0.127322, 0.247322, 0.637322, and 0.757322, M19–M22 were identified as 2′-hydroxylated farrerol-5-O-glucuronide, 3′-hydroxylated farrerol-5-O-glucuronide, 2′-hydroxylated farrerol-7-O-glucuronide and 3′-hydroxylated farrerol-7-O-glucuronide, respectively.

M23 and M24 (C_23_H_24_O_10_, error ≤ 2.5 ppm) were eluted at 8.85 and 9.03 min, and yielded deprotonated ions with *m/z* 459.1308 and 459.1286 [176 Da (C_6_H_8_O_6_) heavier than the ion produced from M11], respectively. Product ions with *m/z* 441.1175 and 283.0897 were formed via loss of H_2_O and C_6_H_8_O_6_, which was indicative of deoxygenation and glucuronide conjugation. Additionally, an important ion with *m/z* 189.0565 was produced by C2–C1′ bond cleavage and had Δ = −16 Da (O), which suggested that deoxygenation took place in ring A. Moreover, two conspicuous ions with *m/z* 295.0971 and 119.0510 were formed. The former ion was produced by the RDA reaction and had Δ = 176 Da. The ion with *m/z* 119.0510 was generated by loss of C_6_H_8_O_6_ from the ion with *m/z* 295.0482, as in the case of the farrerol-derived ion with *m/z* 119.0493. These findings implied that glucuronide conjugation occurred at ring B and allowed M23 (cLog P = 1.45664) and M24 (cLog P = 1.96664) to be identified as 5-dehydroxylated farrerol-4′-*O*-glucuronide and 7-dehydroxylated farrerol-4′-*O*-glucuronide, respectively.

M25–M27 were eluted at 5.24, 5.85, and 6.13 min (Figure 4), yielded deprotonated ions with *m/z* 651.1576, 651.1578, and 651.1588, and featured cLog P values of −1.33398, −1.13098, and −0.62098, respectively. The composition of these isomers was determined as C_29_H_32_O_17_ (error ≤ 3.3 ppm), Δ = 352 Da (2C_6_H_8_O_6_). The two characteristic product ions with *m/z* 475.1244 and 299.0919 were produced by sequential loss of C_6_H_8_O_6_, which suggested that M25–M27 were diglucuronide conjugates of farrerol [31].

M28–M30 were eluted at 5.09, 7.64, and 8.38 min, featured cLog P values of 0.844322, 1.31755, and 1.35432, and were detected at *m/z* 475.1242, 475.1253, and 475.1260, respectively, The above deprotonated ions had Δ = 176 Da (C_6_H_8_O_6_), which was indicative of glucuronide conjugation [23]. The product ions of M28–M30 with *m/z* 429.2053 and 299.0935 were formed by loss of H_2_O + CO and C_6_H_8_O_6_, respectively, while ions with *m/z* 179.0351 and 119.0497 were ascribed to the loss of C_6_H_8_O_6_ followed by the RDA reaction. The composition of M28–M30 was determined as C_23_H_26_O_10_ (error ≤ 3.0 ppm).

M31–M33 (C_23_H_26_O_10_; error ≤ 3.3 ppm) were eluted at 6.38, 6.40 and 7.03 min, featured cLog P values of 1.32317, 1.7964, and 1.83317, and yielded deprotonated ions with *m/z* 461.1438, 461.1441, and 461.1452 (Δ = 162 Da (C_6_H_10_O_5_)), respectively. Product ions with *m/z* 443.1360 and 299.0894 were generated by loss of CO and C_6_H_10_O_5_, which suggested that M31–M33 were glucose conjugation metabolites. Ions with *m/z* 179.0306 and 119.0515 were formed through loss of C_6_H_10_O_5_ and the RDA reaction, and were identical to ions with *m/z* 179.0333 and 119.0493 observed for M0.

M34–M36 (C_17_H_16_O_8_S, error ≤ ±1.8 ppm) were eluted at 6.57, 7.50, and 8.26 min, featured cLog P values of 1.25962, 1.73285, and 1.76962, and yielded deprotonated ions with *m/z* 379.0495, 379.0500 and 379.0495, respectively (Δ = 80 Da (SO_3_)). Numerous product ions were observed, e.g., those with *m/z* 299.0914, 205.0488, 179.0336, and 119.0493 [30]. The ion with *m/z* 299.0914 was produced by loss of SO_3_, as in the case of deprotonated farrerol, which was indicative of sulfate conjugation. Ions with *m/z* 205.0488, 179.0336, and 119.0493 were formed through C2–C1′ bond cleavage and the RDA reaction after loss of SO_3_.

M37–M39 (C_18_H_18_O_5_; error ≤ ±1.7 ppm) were eluted at 8.09, 16.84 and 11.44 min, featured cLog P values of 3.40562, 3.91562, and 3.87885, and yielded deprotonated ions with *m/z* 313.1086, 313.1078, and 313.1079, respectively (Δ = 14 Da (CH_2_)). Two major product ions with *m/z* 298 and 269 were respectively produced by loss of CH_3_ and CO_2_, which was indicative of methylation [23]. Ions with *m/z* 219.0216, 193.0147, and 119.0485, observed for M37 and M38, were produced by C2–C1′ bond cleavage and the RDA reaction, i.e., methylation took place at ring A. Thus, M37 and M38 were identified as 5-methoxyfarrerol and 7-methoxyfarrerol, respectively. The ESI-MS^2^ spectrum of M39 featured ions with *m/z* 179.0338 and 133.0296 formed through the RDA reaction. The observation of the latter ion indicated that ring B was methylated. So, M39 was identified as 4′-methoxyfarrerol.

M40 was eluted at 11.53 min and yielded a deprotonated ion with *m/z* 341.1029 (Δ = 42 Da (C_2_H_2_O)). The corresponding secondary ions were investigated by TOF-MS/MS. Product ions with *m/z* 323.1861 and 297.2049 were produced by loss of H_2_O and CO_2_, respectively, while the ion with *m/z* 295.1910 was formed via loss of CO from the ion with *m/z* 323.1861. Additionally, C2–C1′ bond cleavage and the RDA reaction yielded three ions with *m/z* 205.0479, 179.0351, and 161.0608 [31]. Based on the above, M40 (C_19_H_18_O_6_; error = −0.6 ppm) was identified as the product of farrerol-4′-*O*-N-acetylation.

M41 and M42 (C_22_H_23_NO_8_S; (error ≤ 2.4 ppm) were eluted at 9.03 and 9.50 min and yielded deprotonated ions with *m/z* 460.1062 and 460.1061, respectively (Δ = 161 Da (C_5_H_7_NO_3_S)), which was indicative of N-acetylcysteine conjugate formation. Product ions with *m/z* 297. 0769 (C_17_H_13_O_5_^−^) and 162.0226 (C_5_H_8_NO_3_S^−^) were formed via loss of C_5_H_9_NO_3_S and C_17_H_16_O_5_, respectively, which further supported the above conclusion. However, the exact identities of M41 and M42 remained undetermined, as these compounds featured identical cLog P values of −0.41386.

### 2.5. Metabolic Pathways of Farrerol

**Figure 6:**
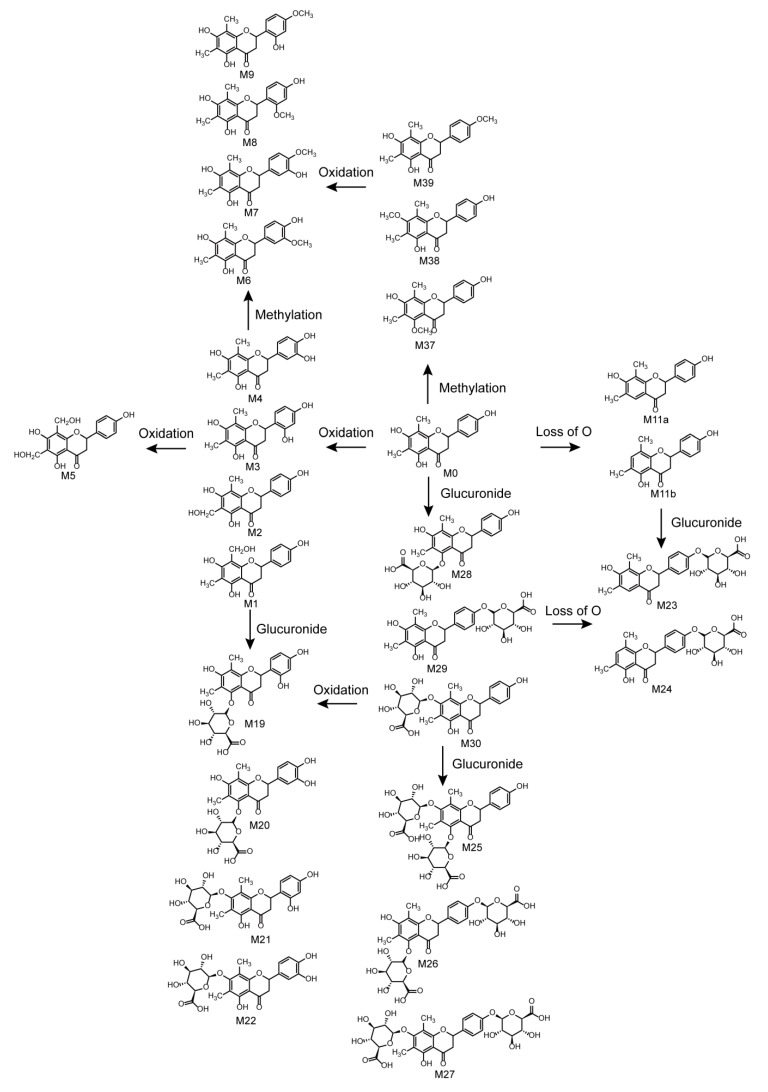

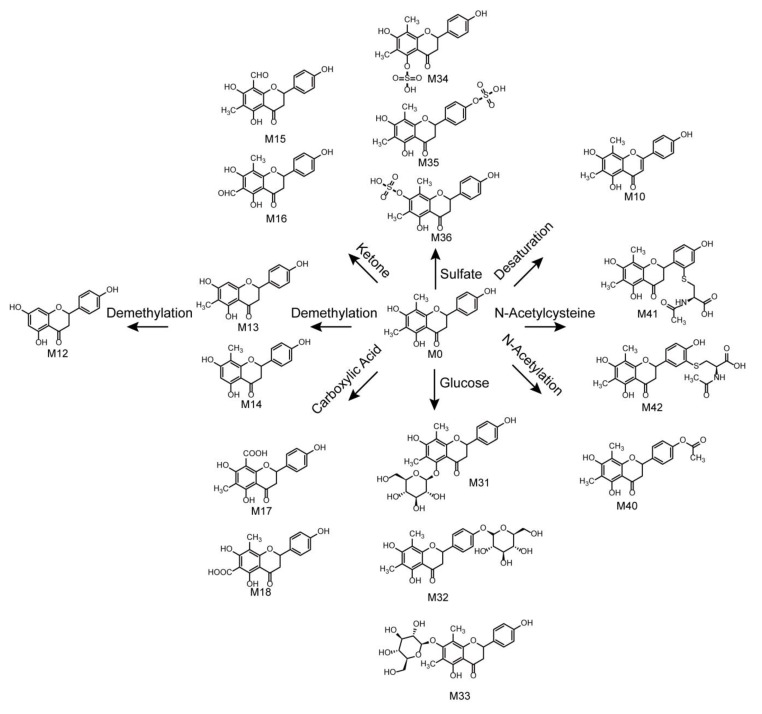
Metabolic pathways of farrerol.

In total, 42 farrerol in vivo metabolites and 15 farrerol in vitro metabolites were identified with the corresponding metabolic pathways summarized in Figure 6. Thus, this work presents the first-time systematic identification of farrerol metabolites and summarizes the related metabolic pathways. The metabolic reactions corresponded to oxidation, reduction, and conjugation. Among them, oxidation, deoxygenation, and demethylation were identified as phase I reactions, while glucose conjugation, glucuronide conjugation, sulfate conjugation, methylation, *N*-acetylation, and *N*-acetylcysteine conjugation were identified as phase II reactions. Moreover, we observed the occurrence of some associative reactions such as oxidation + glucuronide conjugation, oxidation + methylation, and deoxygenation + glucuronide conjugation. Farrerol metabolism in vivo mainly involved both phase I and phase II reactions, while in vitro metabolism mainly involved phase I reactions. Notably, metabolic pathways in vivo were more abundant than those in vitro.

## 3. Discussion

### 3.1. Optimizations of Liquid Conditions and Mass Conditions

To obtain a better chromatographic separation in the appropriate time, the elution duration was set as 25 min, and two elution gradients was set as follows: (i) 10 to 20% B from 0 to 2 min, 20 to 53% B from 2 to 15 min, 53 to 95% B from 15 to 20 min, and 95% B from 20 to 25 min and (ii) 10 to 20% B from 0 to 2 min, 20 to 33% B from 2 to 15 min, 53 to 95% B from 15 to 20 min, and 95% B from 20 to 25 min for analysis. The results showed that the former elution gradient was better. For optimization of mass conditions, online full-scan data were respectively acquired by UHPLC-Q-TOF-MS/MS in positive and negative ESI modes. The stronger response was obtained in negative ESI mode.

### 3.2. Hypotheses of Possible Enzymes

Cytochrome P450 enzymes (CYPs) constitute a major enzyme family and are responsible for phase I metabolism of about 80% endogenous and exogenous substances [32]. The subtypes of CYP1A2, CYP2A6, and CYP2B6 play a vital role in the oxidation of flavanone and flavone [33]. Among them, CYP2A6 has high activities in oxidizing flavanone to form 2′-hydroxyflavanones and flavone, such as metabolites M3 and M10. Additionally, the major metabolic enzymes, for instance, UDP-glucuronosyltransferases (UGTs) sulfotransferase and transmethylase, are involved in phase II metabolism [31]. Moreover, UGTs primarily participate in the glucuronidation of flavanones and flavones. UGT1A1 and UGT1A9 have unique characteristics in catalyzing the glucuronidation at 5-position of flavones, and UGT1A9 and UGT1A10 are responsible for producing the glucuronidation metabolites with hydroxyl groups at the B ring [27]. UGT1A1 and UGT1A9 could involve in formation of metabolites M19, M25, M26 and M28. Beyond that, UGT1A9 and UGT1A10 might catalyze the products of metabolites M20, M23, M24, M27 and M29. The results contributed to understanding the biological transformation of farrerol in vivo.

### 3.3. Semi-quantitative Analysis

**Figure 7:**
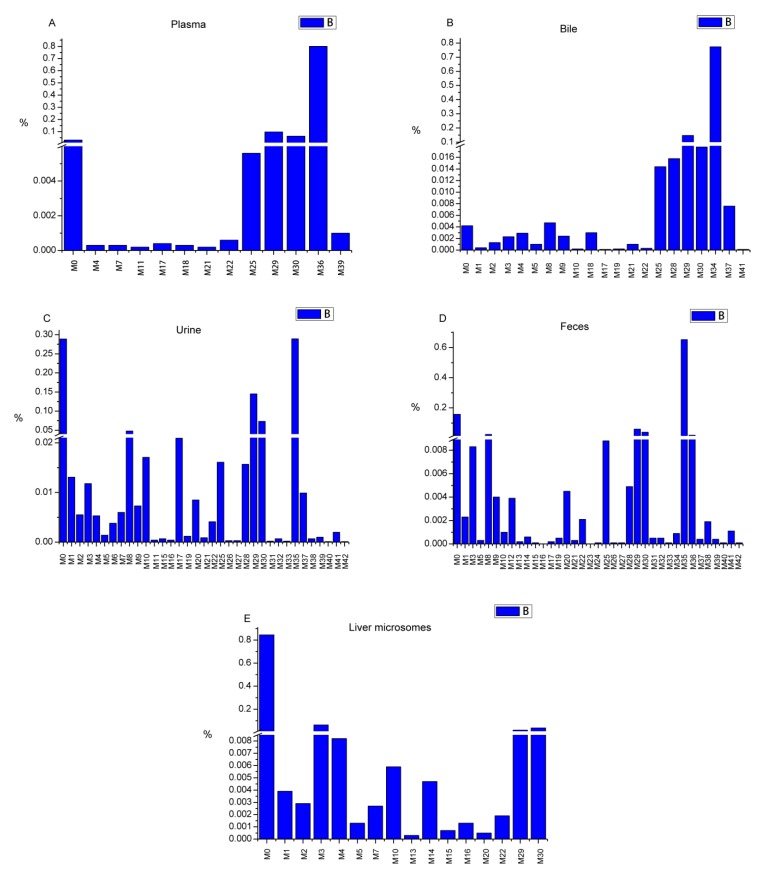
The relative contents of metabolites (RCM) in each sample type (A: RCM in rat plasma sample, B: RCM in rat bile sample, C: RCM in rat urine sample, D: RCM in rat feces sample, E: RCM in rat liver microsome sample).

Area normalization was used for the semi-quantitative analysis of farrerol and its metabolites in all cases [23]. All samples from the same groups were mixed together for analysis. The relative content of each metabolite was calculated by normalizing the area of the metabolite peak by the combined area of farrerol and metabolite peaks. The related data are presented in Table S1. The relative contents of metabolites in each sample type were illustrated in Figure 7. Farrerol was detected in all in vivo and in vitro samples (3.12, 0.42, 28.9, 15.77, and 84.44% in blood, bile, urine, feces, and liver microsomes, respectively). The biological efficiency of farrerol in vivo was higher than that in vitro. Sulfate conjugates, detected only in vivo, were widely metabolized and were the most abundant species in all samples (79.99% in plasma, 77.28% in bile, 28.94% in urine, and 67.19% in feces). Additionally, glucuronide metabolites were produced both in vivo and in vitro, featuring contents of 15.98% in plasma, 18.12% in bile, 23.38% in urine, 10.34% in feces, and 5.72% in liver microsomes. Oxidation metabolites were mainly formed in liver microsomes, where their total content amounted to 7.91%, but were also detected in in vivo samples at lower contents. Other metabolites were observed in vivo and in vitro at lower contents. Thus, we concluded that farrerol is mainly metabolized by conjugation in vivo, while it is largely present in its pristine form in vitro, with a small amount present as oxidation products.

### 3.4. Metabolite Analysis

#### 3.4.1. Comparison of In Vivo and In Vitro Metabolism

Drug metabolism is important for the study of drug safety and toxicity, and therefore plays a vital role in drug discovery and clinical use [18]. The use of rats for in vivo metabolism investigations has been proven to be feasible and efficient [24,25,26], while liver microsomes are well suited for metabolite identification, metabolite preparation, and toxicity research in vitro [19,27]. Herein, 42 farrerol in vivo metabolites and 15 farrerol in vitro metabolites were identified. In vivo, farrerol metabolites were predominantly detected in urine (34 metabolites) and feces (36 metabolites). Additionally, 12 metabolites were characterized in plasma, and 20 metabolites were recognized in bile. In vitro, 15 metabolites were detected in rat liver microsomes. Notably, 18 metabolic pathways were determined in vivo, while only eight metabolic reactions were observed in vitro. Oxidation, desaturation, demethylation, methylation, and glucuronide conjugation were observed both in vivo and in vitro, which confirms the validity of using liver microsomes as an in vitro model. Certain conjugation reactions such as sulfate and *N*-acetylcysteine conjugation were only observed in vivo, which implied that in vivo farrerol metabolism is more complex than that in vitro and highlighted the importance of in vivo metabolism studies.

#### 3.4.2. Reference to Previous Studies

A previous work describes the absorption, distribution, biotransformation, and excretion of farrerol in rats [20]. After oral administration, >70% of farrerol was slowly absorbed and quickly metabolized in vivo. The level of drug distribution in vivo and the amount of prototype drug excreted with urine were low. The results of our semi-quantitative analysis demonstrate that farrerol is widely converted to sulfate (28.94%) and glucuronide (23.38%) conjugates, with the content of residual farrerol being as low as 28.91%. These findings are consistent with those of a previous study, where glucuronide metabolites were detected in urine [20]. Likewise, glucuronide metabolites were also detected in urine by UHPLC-MS in the present work. With respect to pharmacokinetic and bioavailability studies of farrerol, a sensitive LC-MS method developed and validated for the quantitation of farrerol in rat plasma revealed that farrerol is rapidly absorbed and slowly eliminated in rats when administered orally, but is quickly eliminated when administered intravenously [34]. These results provide a theoretical foundation for the study of farrerol metabolites in plasma.

#### 3.4.3. Metabolic Activities of Farrerol

According to previous reports, RD is rich in flavonoids and phenolics [8]. Flavonoids exhibit anti-inflammatory, anti-bacterial, and anti-oxidant activity and can be used for vasodilation and myocardial preservation, thus having the potential to be developed into drugs for cardiovascular disease treatment [35]. Some studies indicated that phenols exhibit anti-bacterial, anti-fungal, anti-inflammatory, anti-oxidant, and styptic activities [8]. Farrerol, a characteristic component of RD, has been developed as an antibechic for clinical use. Studies of metabolite activity are important for the discovery of new pharmacologically active species, as exemplified by the case of M12 (naringenin), which is a naturally occurring compound with a large range of pharmacological (anti-bacterial, anti-immunogenic, anti-obesity, anti-cancer, etc.) activities [36,37,38,39]. All these metabolite activities could open up new avenues for the clinical use of farrerol. All in all, the identification of farrerol metabolites is expected to (i) deepen our understanding of the pharmacological action mechanism of farrerol to ensure the safe use of this flavonoid and (ii) help to discover new pharmacological activities for providing a basis for clinical use.

## 4. Materials and Methods

### 4.1. Chemicals and Materials

Farrerol (>99.8%) was purchased from Chengdu DeSiTe Biological Technology Co., Ltd. (Chengdu, China). Rat liver microsomes were prepared in the Department of Pharmaceutical Analysis of the School of Pharmacy, Hebei Medical University. β-Nicotinamide adenine dinucleotide phosphate (β-NADPH), uridine 5′-diphosphoglucuronic acid trisodium salt (UDPGA), alamethicin, MgCl_2_, and phosphate buffer saline (PBS, pH 7.4) were obtained from BD Biosciences (Woburn, MA, USA). HPLC-grade acetonitrile was obtained from Fisher Scientific (Waltham, MA, USA). HPLC-grade formic acid was supplied by Fisher Scientific (Fair Lawn, NJ, USA). Purified water was procured from Wahaha Group Co., Ltd. (Hangzhou, China). The needles were supplied by Suzhou Suhang Technology Equipment Co., Ltd. (Suzhou, China). The metabolic cages were acquired from Shenzhen Biovano Technology Co., Ltd. (Shenzhen, China). The centrifugation and the ultrasound were performed with Eppendorf Centrifuge 5424 R (Eppendorf AG, Hamburg, Germany) and an E180H ultrasonic cleaner (Elma, Singen, Germany), respectively.

### 4.2. Instruments and Conditions

A UHPLC system (Shimadzu Corp., Kyoto, Japan) equipped with an autosampler, a solvent binary delivery system, and a column oven was connected to a quadrupole TOF 5600 system for analysis.

Chromatographic analysis was performed on a Poroshell 120 EC-C18 (2.1 × 100 mm, 2.7 μm) column (Agilent, Santa Clara, CA, USA) using 0.1 vol% formic acid-water (A) and acetonitrile (B) as mobile phase (flow rate = 0.3 mL/min) components. The column oven was maintained at 40 °C for chromatographic separation. After a series of optimizations, the elution gradient was set as follows: 10 to 20% B from 0 to 2 min, 20 to 53% B from 2 to 15 min, 53 to 95% B from 15 to 20 min, and 95% B from 20 to 25 min. Subsequently, the mobile phase composition was brought back to 10% B over 1 min and maintained for 5 min to equilibrate the column. The injection volume was set to 3 μL.

A quadrupole TOF 5600 system (AB Sciex, Framingham, MA, USA) with Duo-Spray^TM^ ion sources was used for accurate MS and MS^2^ data acquisition. As the metabolite signal intensity in negative-ion ESI mode was stronger than that in the positive-ion mode, all samples were analyzed in the former mode. The MS/MS parameters were as follows: curtain gas pressure = 35 psi; nebulizer gas (gas 1) pressure = 55 psi; heater gas (gas 2) pressure = 55 psi, declustering potential = −60 eV, collision energy = −33 eV, collision energy spread = −15 eV, ion spray voltage = −4.5 kV, turbo spray temperature = 550 °C. A calibration delivery system was utilized for the automatic regulation of MS and MS^2^.

### 4.3. Animals and Drug Administration

Male Sprague Dawley rats (220–240 g) provided by the Experimental Animals Center of Hebei Medical University (Shijiazhuang, China) were housed in a standard environment with a temperature of 25 °C, a relative humidity of 50 ± 20%, a 12-h dark/light cycle, and *ad libitum* feeding for a week. The 18 rats were divided into six groups (groups 1, 3, 5: blood experimental group, bile experimental group, urine and feces experimental group; groups 2, 4, 6: blood blank group, bile blank group and urine and feces blank group) and fasted for 12 h before operation. Farrerol powder was suspended in 0.5 wt% aqueous carboxymethyl cellulose sodium (CMC-Na) and intragastrically administered to experimental groups at a dose of 20 mg/kg [34], while an equivalent dose of CMC-Na solution was given to blank groups. All experiments complied with the Guide for the Care and Use of Laboratory Animals of the U.S. National Institutes of Health.

### 4.4. In Vivo Sample Collection and Pretreatment

For plasma sampling, blood (~200 μL) was collected from the orbital vein at 0.083, 0.25, 1, 2, 3, 5, 7, 9, 12, and 24 h after administration [34]. The specimens were centrifuged at 4,500 rpm for 5 min to obtain plasma samples, which were subsequently mixed and stored at −80 °C.

For bile sampling, rats in experimental group 3 and blank group 4 were anesthetized by intraperitoneal injection of 20 wt% urethane solution (1.5–2 mL) after dosing [24]. Bile was collected using tubes inserted into rat bile ducts via abdominal incision surgery. Sampling was performed within the time periods of 1–2, 2–5, 5–8, 8–12, and 12–24 h after administration. Finally, all collected samples were combined and cryopreserved at −80 °C.

For urine and feces sampling, each rat in experimental group 5 and blank group 6 was placed into a clean metabolic cage after dosing [30]. Urine and feces samples were gathered every 6 h for 72 h. After collection, the samples were combined (separately) and frozen at −80 °C.

The mixed plasma, bile, and urines samples were separately vortexed with three times the volume of acetonitrile, centrifuged at 14,000 rpm for 10 min, and the obtained supernatants were blown dry with N_2_. Naturally dried feces were pulverized and extracted with three times the volume of acetonitrile upon 30-min ultrasonication. The extracts were centrifuged at 6,000 rpm for 10 min, and the supernatants were blown dry with N_2_. The dry residues were re-dissolved in 200 μL acetonitrile for further analysis [23,31].

### 4.5. In Vitro Sample Incubation

A typical phase I incubation system (200 μL final volume) comprised 50 μmol/L farrerol, 0.8 mg/mL microsomal protein, and 2.7 mmol/L MgCl_2_ [30]. The incubation system was processed in 10 × PBS buffer and pre-incubated for 5 min at 37 °C in an incubator oscillator. The system was then supplemented with 1.3 mmol/L NADPH, incubated for 60 min, and then treated with 1 mL acetonitrile to terminate incubation. The mixture was vortexed for 3 min, centrifuged at 14,000 rpm for 10 min, and the organic phase was collected and blown dry with N_2_

A classic incubation mixture (total volume = 200 μL) comprising 50 μmol/L farrerol, 0.8 mg/mL microsomal protein, 2.7 mmol/L MgCl_2_, and 20 μg/mL alamethicin was used for the cascade reaction [27]. As in the case above, incubation was carried out in 10× PBS buffer, and pre-incubation was performed for 5 min at 37 °C in an incubator shaker. Incubation was started by addition of 1.3 mmol/L NADPH and 2.0 mmol/L UDPGA, lasted for 120 min (at 37 °C), and was terminated by addition of 1 mL acetonitrile [27]. The obtained mixtures were vortexed for 3 min and centrifuged at 14,000 rpm for 10 min. The organic layers were collected and blown dry with N_2_

All residues were re-dissolved in 100 μL acetonitrile for analysis. Additionally, in the case of in vitro metabolism, one blank group, one control group, and three sample groups were set up to ensure experiment reliability and authenticity. Blank samples were incubated in the absence of farrerol, which was substituted by an equal amount of acetonitrile, and control samples were incubated in the absence of NADPH or UDPGA. All samples were processed in an identical way [40].

## 5. Conclusions

Herein, a rapid and efficient method combining UHPLC-Q-TOF-MS/MS and multiple data processing techniques was established to roundly screen and systematically identify farrerol metabolites in rats and rat microsomes. In total, 42 metabolites were identified in rats (12 metabolites in plasma, 20 metabolites in bile, 34 metabolites in urine, and 36 metabolites in feces) and 15 metabolites were identified in rat liver microsomes. The major metabolic reactions were oxidation, deoxygenation, demethylation, glucuronide conjugation, and sulfate conjugation, while other less important pathways included ketonization, carboxylic acid formation, glucose conjugation, methylation, *N*-acetylation, and *N*-acetylcysteine conjugation. Moreover, 18 kinds of metabolic pathways were identified, and detailed metabolic profiles of farrerol were established. Thus, this work helps to elucidate the potential pharmacodynamics forms of farrerol, is expected to benefit further investigations of farrerol safety and efficacy, and offers reasonable guidelines for the intake of farrerol-containing drugs and foods. The active metabolites need to be further investigated to develop new clinical applications for disease treatment.

## Figures and Tables

**Table 1:** Summary of phase I and phase II in vivo and in vitro metabolites of farrerol.

Metabolite ID	Reaction	Molecular Formula	*m/z*	Error(ppm)	t_R_(min)	MS/MS Fragments	Clog P	Plasma	Bile	Urine	Feces	RLM
M1	Oxidation	C_17_H_16_O_6_	315.0874	0	4.75	297.0751, 269.2101, 195.0294, 119.0500	1.75585	-*^b^*	+	+*^a^*	+	+
M2	Oxidation	C_17_H_16_O_6_	315.0874	0	5.69	297.0748, 269.0793, 195.0283, 119.0496	1.75585	-	+	+	-	+
M3	Oxidation	C_17_H_16_O_6_	315.0875	0.3	11.65	269.2466, 179.0352, 135.0456	2.57585	-	+	+	+	+
M4	Oxidation	C_17_H_16_O_6_	315.0876	0.6	12.62	269.2126, 179.0354, 135.0455	2.69585	+	+	+	-	+
M5	Dioxidation	C_17_H_16_O_7_	331.0829	1.8	12.84	313.0818, 287.0920, 211.0307, 119.0489	0.218852	-	+	+	+	+
M6	Oxidation and Methylation	C_18_H_18_O_6_	329.1030	−0.1	5.04	314.0789, 283.2643, 205.0482, 179.0341, 149.0591	3.14205	-	+	+	-	-
M7	Oxidation and Methylation	C_18_H_18_O_6_	329.1029	−0.6	6.70	314.0769, 283.2606, 205.0480, 179.0331, 149.0587	3.14205	+	+	+	-	+
M8	Oxidation and Methylation	C_18_H_18_O_6_	329.1040	2.9	12.78	314.0762, 283.2602, 205.0476, 179.0328, 149.0584	3.34205	-	-	+	+	-
M9	Oxidation and Methylation	C_18_H_18_O_6_	329.1031	0.1	13.06	314.0769, 283.2611, 205.0480, 179.0331, 149.0587	3.39205	-	-	+	+	-
M10	Desaturation	C_17_H_14_O_5_	297.0776	2.6	12.20	253.0848, 238.0614, 179.0330, 117.0337	3.75329	-	+	+	+	+
M11a*^c^*	Loss of O	C_17_H_16_O_4_	283.0970	−2	4.20	268.0719, 239.0775,163.0407, 119.0501,	3.43194	+	-	-	-	-
M11b*^c^*							3.94194					
M12	Bisdemethylation	C_15_H_12_O_5_	271.0616	1.6	4.46	227.0357, 225.1065, 177.0462, 151.0039	2.44485	-	-	+	+	-
M13	Demethylation	C_16_H_14_O_5_	285.0765	−1.1	5.38	267.1822, 239.1296, 191.0344, 165.0200, 119.0511	2.84385	-	-	-	+	+
M14	Demethylation	C_16_H_14_O_5_	285.0768	−0.1	12.24	267.1962, 239.1272, 191.0365, 165.0189, 119.0496	2.89385	-	-	-	+	+
M15	Ketone Formation	C_17_H_14_O_6_	313.0714	0.4	7.87	285.0760, 269.0800, 193.0121, 119.0495	2.50612	-	-	+	+	+
M16	Ketone Formation	C_17_H_14_O_6_	313.0723	−1.3	10.85	285.0761, 269.0811, 193.0495, 119.0480	2.92612	-	-	+	+	+
M17	Demethylation to carboxylic acid	C_17_H_14_O_7_	329.0658	−2.7	4.89	311.0566, 283.2634, 209.0093, 119.0484	2.86826	+	+	+	+	-
M18	Demethylation to carboxylic acid	C_17_H_14_O_7_	329.0662	−1.4	7.90	311.0546, 283.2613, 209.0385, 119.0473	3.54826	+	+	-	-	-
M19	Oxidation and glucuronide conjugation	C_23_H_24_O_12_	491.1193	−0.4	5.57	355.0660, 315.0866, 179.0341, 135.0444	0.127322	-	+	+	+	-
M20	Oxidation and glucuronide conjugation	C_23_H_24_O_12_	491.1192	−0.6	6.46	355.0870, 315.0874, 179.0331, 135.0110	0.247322	-	-	+	+	+
M21	Oxidation and glucuronide conjugation	C_23_H_24_O_12_	491.1195	−0.1	7.62	355.0872, 315.0870, 179.0335, 135.0439	0.637322	+	+	+	+	-
M22	Oxidation and Glucuronide Conjugation	C_23_H_24_O_12_	491.1194	−0.2	8.09	355.0867, 315.0874, 179.0343, 135.0448	0.757322	+	+	+	+	+
M23	Loss of O+glucuronidation	C_23_H_24_O_10_	459.1308	2.5	8.85	441.1175, 295.0971, 283.0897, 189.0565, 119.0510	1.45664	-	-	-	+	-
M24	Loss of O+glucuronidation	C_23_H_24_O_10_	459.1286	−2.3	9.03	441.1155, 295.0820, 283.0891, 189.0555, 119.0522	1.96664	-	-	-	+	-
M25	Bisglucuronide conjugation	C_29_H_32_O_17_	651.1576	1.4	5.24	475.1244, 299.0919, 179.0337	−1.33398	+	+	+	+	-
M26	Bisglucuronide conjugation	C_29_H_32_O_17_	651.1578	1.8	5.85	475.1240, 299.0913, 179.0331	−1.13098	-	-	+	+	-
M27	Bisglucuronide conjugation	C_29_H_32_O_17_	651.1588	3.3	6.13	475.1240, 299.0910, 179.0325	−0.62098	-	-	+	+	-
M28	Glucuronidation conjugation	C_23_H_24_O_11_	475.1242	−0.7	5.09	429.2053, 299.0935, 271.0566, 179.0351, 119.0497	0.844322	-	+	+	+	-
M29	Glucuronidation conjugation	C_23_H_24_O_11_	475.1253	1.4	7.64	429.2086, 299.0910, 271.0626, 179.0329, 119.0486	1.31755	+	+	+	+	+
M30	Glucuronidation conjugation	C_23_H_24_O_11_	475.1260	3.0	8.38	429.1939, 299.0971, 271.0566, 179.0331, 119.0488	1.35432	+	+	+	+	+
M31	Glucose conjugation	C_23_H_26_O_10_	461.1438	−3.3	6.38	443.1360, 299.0894, 285.1122, 205.0585, 179.0306, 119.0515	1.32317	-	-	+	+	-
M32	Glucose conjugation	C_23_H_26_O_10_	461.1441	−2.7	6.40	443.1351, 299.0889, 285.1121, 205.0575, 179.0336, 119.0525	1.7964	-	-	+	+	-
M33	Glucose conjugation	C_23_H_26_O_10_	461.1452	−0.3	7.03	443.1353, 299.0874, 285.1134, 205.0565, 179.0332, 119.0521	1.83317	-	-	+	+	-
M34	Sulfate conjugation	C_17_H_16_O_8_S	379.0495	0.4	6.57	374.0495, 299.0914, 205.0488, 179.0336, 119.0493	1.25962	-	+	-	+	-
M35	Sulfate conjugation	C_17_H_16_O_8_S	379.0500	1.8	7.50	374.0500, 299.0879, 205.0476, 179.0324, 119.0485	1.73285	-	-	+	+	-
M36	Sulfate conjugation	C_17_H_16_O_8_S	379.0495	0.6	8.26	374.0495, 299.0897, 205.0476, 179.0334, 119.0487	1.76962	+	-	-	+	-
M37	Methylation	C_18_H_18_O_5_	313.1086	1.7	8.09	298.0839, 271.0984, 269.1748, 219.0216, 193.0147, 119.0485	3.40562	-	+	+	+	-
M38	Methylation	C_18_H_18_O_5_	313.1078	−1.1	16.84	298.0847, 271.0974,269.1737, 219.0223, 193.0156, 119.0489	3.91562	-	-	+	+	-
M39	Methylation	C_18_H_18_O_5_	313.1079	−0.7	11.44	298.0841, 269.1763, 267.1589, 179.0338, 133.0296	3.87885	+	-	+	+	-
M40	N-acetylation	C_19_H_18_O_6_	341.1029	−0.6	11.53	323.1861, 297.2049, 295.1910, 205.0479, 179.0351, 135.0442	3.30885	-	-	+	+	-
M41	N-acetylcysteine conjugation	C_22_H_23_NO_8_S	460.1062	−2.2	9.03	297.0769, 177.0185, 162.0226, 119.0497	−0.41386	-	+	+	+	-
M42	N-acetylcysteine conjugation	C_22_H_23_NO_8_S	460.1061	−2.4	9.50	297.0779, 177.0179, 162.0231, 119.0487	−0.41386	-	-	+	+	-

+*^a^*: detected; -*^b^*: undetected; a*^c^*, b*^c^*: possible structure; RLM: Rat liver microsomes.

## Supplementary Materials

The following are available online at https://www.mdpi.com/1420-3049/24/19/3470/s1, Table S1: The areas and relative contents of metabolites of farrerol in vivo and in vitro.
