# Supplementary material for: A Complete Study of Farrerol Metabolites Produced In Vivo and In Vitro
Source: Molecules. 2019 Sep 24;24(19):3470. doi: 10.3390/molecules24193470 (PMC6804004; doi:10.3390/molecules24193470)
Supplement: Supplementary file 1 [file molecules-24-03470-s001.pdf]

Table S1 The areas and relative contents of metabolites of farrerol *in vivo* and *in vitro*.

ID	*In vivo*								*In vitro*	
	Plasma		Bile		Urine		Feces		RLM	
	area	%content	area	%content	area	%content	area	%content	area	%content
M0	1.85E+06	3.12%	2.36E+06	0.42%	9.25E+07	28.91%	1.49E+08	15.77%	6.42E+07	84.44%
M1	-[a]	-	2.39E+05	0.04%	4.18E+06	1.31%	2.18E+06	0.23%	2.96E+05	0.39%
M2	-	-	7.29E+05	0.13%	1.75E+06	0.55%	-	-	2.24E+05	0.29%
M3	-	-	1.28E+06	0.23%	3.78E+06	1.18%	7.81E+06	0.83%	4.87E+06	6.41%
M4	2.04E+04	0.03%	1.65E+06	0.29%	1.70E+06	0.53%	-	-	6.21E+05	0.82%
M5	-	-	5.54E+05	0.10%	4.57E+05	0.14%	2.38E+05	0.03%	9.85E+04	0.13%
M6	-	-	-	-	1.21E+06	0.38%	-	-	-	-
M7	1.94E+04	0.03%	-	-	1.92E+06	0.60%	-	-	2.04E+05	0.27%
M8	-	-	2.65E+06	0.47%	1.53E+07	4.78%	2.26E+07	2.39%	-	-
M9	-	-	1.36E+06	0.24%	2.33E+06	0.73%	3.76E+06	0.40%	-	-
M10	-	-	1.11E+05	0.02%	5.47E+06	1.71%	9.21E+05	0.10%	4.46E+05	0.59%
M11	1.30E+04	0.02%	-	-	1.31E+05	0.04%	-	-	-	-
M12	-	-	-	-	-	-	3.66E+06	0.39%	-	-
M13	-	-	-	-	-	-	2.17E+05	0.02%	2.32E+04	0.03%
M14	-	-	-	-	-	-	5.91E+05	0.06%	3.58E+05	0.47%
M15	-	-	-	-	2.16E+05	0.07%	8.63E+04	0.01%	5.67E+04	0.07%
M16	-	-	-	-	1.25E+05	0.04%	1.37E+04	0.001%	9.64E+04	0.13%
M17	2.57E+04	0.04%	1.66E+06	0.30%	6.68E+06	2.09%	2.16E+05	0.02%	-	-
M18	1.76E+04	0.03%	3.09E+04	0.01%	-	-	-	-	-	-
M19	-	-	9.32E+04	0.02%	3.96E+05	0.12%	4.57E+05	0.05%	-	-
M20	-	-		-	2.71E+06	0.85%	4.29E+06	0.45%	3.72E+04	0.05%
M21	1.44E+04	0.02%	5.54E+05	0.10%	2.89E+05	0.09%	2.87E+05	0.03%	-	-
M22	3.31E+04	0.06%	1.74E+05	0.03%	1.32E+06	0.41%	2.03E+06	0.21%	1.48E+05	0.19%
M23	-	-	-	-	-	-	1.24E+04	0.001%	-	-
M24	-	-	-	-	-	-	4.91E+04	0.01%	-	-
M25	3.33E+05	0.56%	8.06E+06	1.44%	5.15E+06	1.61%	8.27E+06	0.88%	-	-
M26	-	-	-	-	8.39E+04	0.03%	1.08E+05	0.01%	-	-
M27	-	-	-	-	9.15E+04	0.03%	1.30E+05	0.01%	-	-
M28	-	-	8.83E+06	1.58%	5.03E+06	1.57%	4.66E+06	0.49%	-	-
M29	5.76E+06	9.72%	8.27E+07	14.76%	4.64E+07	14.50%	5.60E+07	5.93%	1.45E+06	1.91%
M30	3.71E+06	6.26%	1.00E+07	1.78%	2.34E+07	7.31%	3.70E+07	3.92%	2.90E+06	3.81%
M31	-	-	-	-	5.20E+04	0.02%	4.53E+05	0.05%	-	-
M32	-	-	-	-	2.29E+05	0.07%	5.13E+05	0.05%	-	-
M33	-	-	-	-	6.11E+04	0.02%	1.37E+05	0.01%	-	-
M34	-	-	4.33E+08	77.28%	-	-	8.56E+05	0.09%	-	-

M35	-	-	-	-	9.26E+07	28.94%	6.16E+08	65.20%	-	-
M36	4.74E+07	79.99%	-	-	-	-	1.84E+07	1.95%	-	-
M37	-	-	4.24E+06	0.76%	3.18E+06	0.99%	4.11E+05	0.04%	-	-
M38	-	-	-	-	2.21E+05	0.07%	1.79E+06	0.19%	-	-
M39	5.94E+04	0.10%	-	-	3.29E+05	0.10%	4.22E+05	0.04%	-	-
M40	-	-	-	-	3.09E+04	0.01%	7.26E+04	0.01%	-	-
M41	-	-	5.79E+04	0.01%	6.33E+05	0.20%	1.05E+06	0.11%	-	-
M42	-	-	-	-	3.56E+04	0.01%	7.25E+04	0.01%	-	-
sum	5.93E+07	100.00%	5.60E+08	100.00%	3.20E+08	100.00%	9.45E+08	100.00%	7.60E+07	100.00%

[a]undetected, RLM: Rat liver microsome.
